# Supplementary material for: Optogenetic dissection of mitotic spindle positioning in vivo
Source: eLife. 2018 Aug 15;7:e38198. doi: 10.7554/eLife.38198 (PMC6214656; doi:10.7554/eLife.38198)
Supplement: Supplementary file 1. [file elife-38198-supp1.docx]

| **Supplementary Table 1: genotypes of *C. elegans* used in this study, listed per figure** | | | |
| --- | --- | --- | --- |
| **Figure** | **Strain** | **Genotype** | **Source** |
| **1 b** | SA250 | tjls54(Ppie-1::gfp::tbb-2 + Ppie-1::2xmcherry::tbg-1 + unc-119(+)); Tjls57(Ppie-1::mcherry::his-48 + unc-119(+)) | CGC |
|  | SV1589 | lin-5(he244[co-egfp::lin-5]) II | (Portegijs et al., 2016) |
|  | SV1569 | gpr-1(he238[co-fkbp::co-egfp::gpr-1]) III | (Portegijs et al., 2016) |
|  | SV1803 | dhc-1(he263[egfp::dhc-1]) I | (Schmidt et al., 2017) |
| **1 c** | AZ244 | unc-119(ed3) III; ruls57[Ppie-1::gfp::tbb-2 + unc119(+)] V | CGC |
| **1 d** | AZ244 | unc-119(ed3) III; ruls57[Ppie-1::gfp::tbb-2 + unc119(+)] V | CGC |
|  | SV1485 | lin-5(ev571ts) II; ruls57[Ppie-1::gfp::tbb-2 + unc119(+)] V |  |
| **1 e** | AZ244 | unc-119(ed3) III; ruls57[Ppie-1::gfp::tbb-2 + unc119(+)] V | CGC |
|  | SV2035 | ttTi5605(he312[Ppie-1(long)::frt::glo-mcherry::cep-1(3'UTR)::frt::egl-13nls::glo-cre::cep-1(3'UTR)]) II; ric-8(he280[loxN -741/loxN 4624]) IV; ruls57[Ppie-1::gfp::tbb-2 + unc119(+)] V; Ppie-1::sv40nls::flp(G5D)::egl-13nls::tbb-2(3'UTR) + Pmyo-2::tdtomato + *E. coli* DNA c/w PvuII Ex |  |
|  | SV2087 | ttTi5605(he312[Ppie-1(long)::frt::glo-mcherry::cep-1(3'UTR)::frt::egl-13nls::glo-cre::cep-1(3'UTR)]) II; ruls57[Ppie-1::gfp::tbb-2 + unc119(+)] V; rgs-7(he300[loxP in intron 2 + -CT co-mcherry::co-epdz::tev::loxP::3xflag]) X; Ppie-1::sv40nls::flp(G5D)::egl-13nls::tbb-2(3'UTR) + Pmyo-2::tdtomato + *E. coli* DNA c/w PvuII Ex |  |
|  | SV2088 | ttTi5605(he312[Ppie-1(long)::frt::glo-mcherry::cep-1(3'UTR)::frt::egl-13nls::glo-cre::cep-1(3'UTR)]) II; ric-8(he280[loxN -741/loxN 4624]) IV; ruls57[Ppie-1::gfp::tbb-2 + unc119(+)] V; rgs-7(he300[loxP in intron 2 + -CT co-mCherry::co-epdz::tev::loxP::3xflag]) X; Ppie-1::sv40nls::flp(G5D)::egl-13nls::tbb-2(3'UTR) + Pmyo-2::tdtomato + *E. coli* DNA c/w PvuII Ex |  |
|  |  |  |  |
| **2 b** | SV2061 | ttTi5605(he314[Ppie-1::glo-epdz::mcherry(smu-1)::tbb-2(3'UTR)]) II; cxTi10816(he259[Peft-3::ph::co-egfp::co-lov::tbb-2(3'UTR)]) IV |  |
|  |  |  |  |
| **3 a** | SV2076 | cxTi10816(he259[Peft-3::ph::co-egfp::co-lov::tbb-2(3'UTR)]) IV; ruls57[Ppie-1::gfp::tbb-2 + unc119(+)] V; rgs-7(he290[rgs-7::co-mcherry::co-epdz]) X |  |
|  | SV2062 | par-6(he322[par-6::gfp(smu-1 introns)::glo-lov] I |  |
| **3 b** | SV2074 | ruls57[Ppie-1::gfp::tbb-2 + unc119(+)] V; rgs-7(he290[rgs-7::co-mcherry::co-epdz]) X |  |
|  | SV2076 | CxTi 10816(he259[Peft-3::PH::eGFP::LOV::tbb-2]) IV ; RuIs57 [ Ppie-1::Tub::GFP) V ; rgs-7 (he290[rgs-7::co-mCherry::co-ePDZ]) X |  |
|  | SV2109 | par-6 (he322 [par-6::gfp(smu-1 introns)::glo-lov]) I ; RuIs57 (Ppie-1::Tub::GFP) V |  |
|  | SV2143 | par-6(he322[par-6::gfp(smu-1 introns)::glo-lov] I; ruls57[Ppie-1::gfp::tbb-2 + unc119(+)] V; rgs-7(he290[rgs-7::co-mcherry::co-epdz]) X |  |
| **3 c** | SV2074 | ruls57[Ppie-1::gfp::tbb-2 + unc119(+)] V; rgs-7(he290[rgs-7::co-mcherry::co-epdz]) X |  |
|  | SV2076 | CxTi 10816(he259[Peft-3::PH::eGFP::LOV::tbb-2]) IV ; RuIs57 [ Ppie-1::Tub::GFP) V ; rgs-7 (he290[rgs-7::co-mCherry::co-ePDZ]) X |  |
|  | SV2143 | par-6(he322[par-6::gfp(smu-1 introns)::glo-lov] I; ruls57[Ppie-1::gfp::tbb-2 + unc119(+)] V; rgs-7(he290[rgs-7::co-mcherry::co-epdz]) X |  |
|  |  |  |  |
| **4 a** | SV2147 | ric-8(he339[glo-epdz::mcherry(smu-1 introns)::ric-8]) IV; cxTi10816(he259[Peft-3::ph::co-egfp::co-lov::tbb-2(3'UTR)]) IV; ruls57[Ppie-1::gfp::tbb-2 + unc119(+)] V |  |
| **4 b** | AZ244 | unc-119(ed3) III; ruls57[Ppie-1::gfp::tbb-2 + unc119(+)] V | CGC |
|  | SV2085 | cxTi10816(he259[Peft-3::ph::co-egfp::co-lov::tbb-2(3'UTR)]) IV; ruls57[Ppie-1::gfp::tbb-2 + unc119(+)] V |  |
|  | SV2146 | ric-8(he339[glo-epdz::mcherry(smu-1 introns)::ric-8]) IV; ruls57[Ppie-1::gfp::tbb-2 + unc119(+)] V |  |
|  | SV2147 | ric-8(he339[glo-epdz::mcherry(smu-1 introns)::ric-8]) IV; cxTi10816(he259[Peft-3::ph::co-egfp::co-lov::tbb-2(3'UTR)]) IV; ruls57[Ppie-1::gfp::tbb-2 + unc119(+)] V |  |
|  |  |  |  |
| **5 b** | SV2043 | gpr-1(he301[glo-epdz::mcherry(smu-1 introns)::gpr-1]), gpr-2(he311[Δ7 - 1212]) III; cxTi10816(he259[Peft-3::ph::co-egfp::co-lov::tbb-2(3'UTR)]) IV |  |
| **5 c** | SV2043 | gpr-1(he301[glo-epdz::mcherry(smu-1 introns)::gpr-1]), gpr-2(he311[Δ7 - 1212]) III; cxTi10816(he259[Peft-3::ph::co-egfp::co-lov::tbb-2(3'UTR)]) IV |  |
| **5 d** | SV2038 | gpr-1(he301[glo-epdz::mcherry(smu-1 introns)::gpr-1]), gpr-2(he311[Δ7 - 1212]) III; ruls57[Ppie-1::gfp::tbb-2 + unc119(+)] V |  |
|  | SV2059 | gpr-1(he301[glo-epdz::mcherry(smu-1 introns)::gpr-1]), gpr-2(he311[Δ7 - 1212]) III; cxTi10816(he259[Peft-3::ph::co-egfp::co-lov::tbb-2(3'UTR)]) IV; ruls57[Ppie-1::gfp::tbb-2 + unc119(+)] V |  |
| **5 e** | SV2091 | weIs21[pJA138 (Ppie-1::mcherry::tubulin::pie-1 + unc-119(+)]; unc-119(ed3) III; gpr-1(he301[glo-epdz::mcherry(smu-1 introns)::gpr-1]), gpr-2(he311[Δ7 - 1212]) III |  |
|  | SV2099 | weIs21(pJA138[Ppie-1::mcherry::tubulin::pie-1(3'UTR)]); gpr-1(he301[glo-epdz::mcherry(smu-1 introns)::gpr-1]), gpr-2(he311[Δ7-1212])III; cxTi10816(he259[Peft-3::ph::co-egfp::co-lov::tbb-2(3'UTR)]) IV |  |
| **5 f** | SV2085 | cxTi10816(he259[Peft-3::ph::co-egfp::co-lov::tbb-2(3'UTR)]) IV; ruls57[Ppie-1::gfp::tbb-2 + unc119(+)] V |  |
|  | SV2038 | gpr-1(he301[glo-epdz::mcherry(smu-1 introns)::gpr-1]), gpr-2(he311[Δ7 - 1212]) III; ruls57[Ppie-1::gfp::tbb-2 + unc119(+)] V |  |
|  | SV2059 | gpr-1(he301[glo-epdz::mcherry(smu-1 introns)::gpr-1]), gpr-2(he311[Δ7 - 1212]) III; cxTi10816(he259[Peft-3::ph::co-egfp::co-lov::tbb-2(3'UTR)]) IV; ruls57[Ppie-1::gfp::tbb-2 + unc119(+)] V |  |
| **5 g** | SV2085 | cxTi10816(he259[Peft-3::ph::co-egfp::co-lov::tbb-2(3'UTR)]) IV; ruls57[Ppie-1::gfp::tbb-2 + unc119(+)] V |  |
| **5 h** | SV2085 | cxTi10816(he259[Peft-3::ph::co-egfp::co-lov::tbb-2(3'UTR)]) IV; ruls57[Ppie-1::gfp::tbb-2 + unc119(+)] V |  |
|  | SV2059 | gpr-1(he301[glo-epdz::mcherry(smu-1 introns)::gpr-1]), gpr-2(he311[Δ7 - 1212]) III; cxTi10816(he259[Peft-3::ph::co-egfp::co-lov::tbb-2(3'UTR)]) IV; ruls57[Ppie-1::gfp::tbb-2 + unc119(+)] V |  |
|  |  |  |  |
| **6 b** | SV2090 | dhc-1(he255[epdz::mcherry::dhc-1])/+ I; cxTi10816(he259[Peft-3::ph::co-egfp::co-lov::tbb-2(3'UTR)]) IV; ruls57[Ppie-1::gfp::tbb-2 + unc119(+)] V; |  |
| **6 c** | SV2090 | dhc-1(he255[epdz::mcherry::dhc-1])/+ I; cxTi10816(he259[Peft-3::ph::co-egfp::co-lov::tbb-2(3'UTR)]) IV; ruls57[Ppie-1::gfp::tbb-2 + unc119(+)] V; |  |
| **6 d** | SV2095 | dhc-1(he255[epdz::mcherry::dhc-1]) I; ruls57[Ppie-1::gfp::tbb-2 + unc119(+)] V |  |
|  | SV2090 | dhc-1(he255[epdz::mcherry::dhc-1])/+ I; cxTi10816(he259[Peft-3::ph::co-egfp::co-lov::tbb-2(3'UTR)]) IV; ruls57[Ppie-1::gfp::tbb-2 + unc119(+)] V; |  |
| **6 e** | SV2095 | dhc-1(he255[epdz::mcherry::dhc-1]) I; ruls57[Ppie-1::gfp::tbb-2 + unc119(+)] V |  |
|  | SV2090 | dhc-1(he255[epdz::mcherry::dhc-1])/+ I; cxTi10816(he259[Peft-3::ph::co-egfp::co-lov::tbb-2(3'UTR)]) IV; ruls57[Ppie-1::gfp::tbb-2 + unc119(+)] V; |  |
| **6 f** | SV2095 | dhc-1(he255[epdz::mcherry::dhc-1]) I; ruls57[Ppie-1::gfp::tbb-2 + unc119(+)] V |  |
|  | SV2090 | dhc-1(he255[epdz::mcherry::dhc-1])/+ I; cxTi10816(he259[Peft-3::ph::co-egfp::co-lov::tbb-2(3'UTR)]) IV; ruls57[Ppie-1::gfp::tbb-2 + unc119(+)] V; |  |
|  |  |  |  |
| **7 b** | SV2121 | lin-5(he330[lin-5::glo-epdz::mcherry(smu-1 introns)]) II; cxTi10816(he259[Peft-3::ph::co-egfp::co-lov::tbb-2(3'UTR)]) IV; ruls57[Ppie-1::gfp::tbb-2 + unc119(+)] V |  |
| **7 c** | SV2121 | lin-5(he330[lin-5::glo-epdz::mcherry(smu-1 introns)]) II; cxTi10816(he259[Peft-3::ph::co-egfp::co-lov::tbb-2(3'UTR)]) IV; ruls57[Ppie-1::gfp::tbb-2 + unc119(+)] V |  |
| **7 d** | SV2120 | lin-5(he330[lin-5::glo-epdz::mcherry(smu-1 introns)]) II; ruls57[Ppie-1::gfp::tbb-2 + unc119(+)] V; |  |
|  | SV2121 | lin-5(he330[lin-5::glo-epdz::mcherry(smu-1 introns)]) II; cxTi10816(he259[Peft-3::ph::co-egfp::co-lov::tbb-2(3'UTR)]) IV; ruls57[Ppie-1::gfp::tbb-2 + unc119(+)] V |  |
| **7 e** | SV2120 | lin-5(he330[lin-5::glo-epdz::mcherry(smu-1 introns)]) II; ruls57[Ppie-1::gfp::tbb-2 + unc119(+)] V; |  |
|  | SV2121 | lin-5(he330[lin-5::glo-epdz::mcherry(smu-1 introns)]) II; cxTi10816(he259[Peft-3::ph::co-egfp::co-lov::tbb-2(3'UTR)]) IV; ruls57[Ppie-1::gfp::tbb-2 + unc119(+)] V |  |
| **7 f** | SV2085 | cxTi10816(he259[Peft-3::ph::co-egfp::co-lov::tbb-2(3'UTR)]) IV; ruls57[Ppie-1::gfp::tbb-2 + unc119(+)] V |  |
|  | SV2120 | lin-5(he330[lin-5::glo-epdz::mcherry(smu-1 introns)]) II; ruls57[Ppie-1::gfp::tbb-2 + unc119(+)] V; |  |
|  | SV2121 | lin-5(he330[lin-5::glo-epdz::mcherry(smu-1 introns)]) II; cxTi10816(he259[Peft-3::ph::co-egfp::co-lov::tbb-2(3'UTR)]) IV; ruls57[Ppie-1::gfp::tbb-2 + unc119(+)] V |  |
|  |  |  |  |
| **8 a** | SV2121 | lin-5(he330[lin-5::glo-epdz::mcherry(smu-1 introns)]) II; cxTi10816(he259[Peft-3::ph::co-egfp::co-lov::tbb-2(3'UTR)]) IV; ruls57[Ppie-1::gfp::tbb-2 + unc119(+)] V |  |
| **8 b** | SV2121 | lin-5(he330[lin-5::glo-epdz::mcherry(smu-1 introns)]) II; cxTi10816(he259[Peft-3::ph::co-egfp::co-lov::tbb-2(3'UTR)]) IV; ruls57[Ppie-1::gfp::tbb-2 + unc119(+)] V |  |
| **8 c** | SV2121 | lin-5(he330[lin-5::glo-epdz::mcherry(smu-1 introns)]) II; cxTi10816(he259[Peft-3::ph::co-egfp::co-lov::tbb-2(3'UTR)]) IV; ruls57[Ppie-1::gfp::tbb-2 + unc119(+)] V |  |
|  |  |  |  |
| **Figure 1-figure supplement 2 a** | AZ244 | unc-119(ed3) III; ruls57[Ppie-1::gfp::tbb-2 + unc119(+)] V | CGC |
|  | SV2087 | ttTi5605(he312[Ppie-1(long)::frt::glo-mcherry::cep-1(3'UTR)::frt::egl-13nls::glo-cre::cep-1(3'UTR)]) II; ruls57[Ppie-1::gfp::tbb-2 + unc119(+)] V; rgs-7(he300[loxP in intron 2 + -CT co-mcherry::co-epdz::tev::loxP::3xflag]) X |  |
|  |  | ttTi5605(he312[Ppie-1(long)::frt::glo-mcherry::cep-1(3'UTR)::frt::egl-13nls::glo-cre::cep-1(3'UTR)]) II; ruls57[Ppie-1::gfp::tbb-2 + unc119(+)] V; rgs-7(he300[loxP in intron 2 + -CT co-mcherry::co-epdz::tev::loxP::3xflag]) X; Ppie-1::sv40nls::flp(G5D)::egl-13nls::tbb-2(3'UTR) + Pmyo-2::tdtomato + *E. coli* DNA c/w PvuII Ex |  |
|  | SV2035 | ttTi5605(he312[Ppie-1(long)::frt::glo-mcherry::cep-1(3'UTR)::frt::egl-13nls::glo-cre::cep-1(3'UTR)]) II; ric-8(he280[loxN -741/loxN 4624]) IV; ruls57[Ppie-1::gfp::tbb-2 + unc119(+)] V |  |
|  |  | ttTi5605(he312[Ppie-1(long)::frt::glo-mcherry::cep-1(3'UTR)::frt::egl-13nls::glo-cre::cep-1(3'UTR)]) II; ric-8(he280[loxN -741/loxN 4624]) IV; ruls57[Ppie-1::gfp::tbb-2 + unc119(+)] V; Ppie-1::sv40nls::flp(G5D)::egl-13nls::tbb-2(3'UTR) + Pmyo-2::tdtomato + *E. coli* DNA c/w PvuII Ex |  |
|  | SV2088 | ttTi5605(he312[Ppie-1(long)::frt::glo-mcherry::cep-1(3'UTR)::frt::egl-13nls::glo-cre::cep-1(3'UTR)]) II; ric-8(he280[loxN -741/loxN 4624]) IV; ruls57[Ppie-1::gfp::tbb-2 + unc119(+)] V; rgs-7 (he300 [loxP in intron 2 + -CT co-mCherry::co-epdz::TEV::loxP::3xFlag]) X |  |
|  |  | ttTi5605 (he312 [long Ppie-1::FRT::glo-mCherry::cep-1 3' UTR::FRT::egl-13 NLS::glo-Cre::cep-1 3' UTR]) II ; ric-8 (he280[loxN -741/loxN 4624]) IV ; RuIs57(Ppie-1::GFP::tubulin)V ; rgs-7 (he300 [loxP in intron 2 + -CT co-mCherry::co-epdz::TEV::loxP::3xFlag]) X; Ppie-1::sv40nls::flp(G5D)::egl-13nls::tbb-2(3'UTR) + Pmyo-2::tdtomato + *E. coli* DNA c/w PvuII Ex |  |
| **Figure 1-figure supplement 2 b** | AZ244 | unc-119(ed3) III; ruls57[Ppie-1::gfp::tbb-2 + unc119(+)] V | CGC |
|  | SV1460 | ric-8(md303) I; ruls57[Ppie-1::gfp::tbb-2 + unc119(+)] V |  |
|  |  |  |  |
| **Figure 2-figure supplement 2 a** | LP185 | cpIs25[Pmex-5::mNeonGreen::AraD::2xStrep::tbb-2 3'UTR + unc-119(+)] II; unc-119(ed3) III |  |
|  | LP186 | cpIs26[Pmex-5::mNeonGreen::AraD::2xStrep::tbb-2 3'UTR + unc-119(+)] II; unc-119(ed3) III |  |
|  | LP187 | cpIs27[Pmex-5::mNeonGreen::AraD::2xStrep::tbb-2 3'UTR + unc-119(+)] II; unc-119(ed3) III |  |
|  | LP230 | cpIs36[Pmex-5::mNeonGreen::3xFlag::AraD::tbb-2 3'UTR + unc-119(+)] II; unc-119(ed3) III |  |
|  | LP231 | cpIs37[Pmex-5::mNeonGreen::3xFlag::AraD::tbb-2 3'UTR + unc-119(+)] II; unc-119(ed3) III |  |
|  | LP232 | cpIs38[Pmex-5::mNeonGreen::3xFlag::AraD::tbb-2 3'UTR + unc-119(+)] II; unc-119(ed3) III |  |
|  | LP233 | cpIs39[Pmex-5::mNeonGreen::3xFlag::AraD::tbb-2 3'UTR + unc-119(+)] II; unc-119(ed3) III |  |
| **Figure 2-figure supplement 2 b** | LP185 | cpIs25[Pmex-5::mNeonGreen::AraD::2xStrep::tbb-2 3'UTR + unc-119(+)] II; unc-119(ed3) III |  |
|  | LP186 | cpIs26[Pmex-5::mNeonGreen::AraD::2xStrep::tbb-2 3'UTR + unc-119(+)] II; unc-119(ed3) III |  |
|  | LP187 | cpIs27[Pmex-5::mNeonGreen::AraD::2xStrep::tbb-2 3'UTR + unc-119(+)] II; unc-119(ed3) III |  |
|  | LP230 | cpIs36[Pmex-5::mNeonGreen::3xFlag::AraD::tbb-2 3'UTR + unc-119(+)] II; unc-119(ed3) III |  |
|  | LP231 | cpIs37[Pmex-5::mNeonGreen::3xFlag::AraD::tbb-2 3'UTR + unc-119(+)] II; unc-119(ed3) III |  |
|  | LP232 | cpIs38[Pmex-5::mNeonGreen::3xFlag::AraD::tbb-2 3'UTR + unc-119(+)] II; unc-119(ed3) III |  |
|  | LP233 | cpIs39[Pmex-5::mNeonGreen::3xFlag::AraD::tbb-2 3'UTR + unc-119(+)] II; unc-119(ed3) III |  |
| **Figure 2-figure supplement 3** | LP288 | cpSi22[Pcdk-1::gfp::cdk-1 + unc-119(+)] II; unc-119(ed3) III |  |
|  | LP294 | cpSi28[Pcdk-1::gfp-GLO::cdk-1 + unc-119(+)] II; unc-119(ed3) III |  |
|  |  |  |  |
| **Figure 5-figure supplement 1** | SV1972 | gpr-1(he301[glo-epdz::mcherry(smu-1 introns)::gpr-1]) III |  |
|  | SV2085 | cxTi10816(he259[Peft-3::ph::co-egfp::co-lov::tbb-2(3'UTR)]) IV; ruls57[Ppie-1::gfp::tbb-2 + unc119(+)] V |  |
|  | SV1982 | gpr-1 (he301[glo-epdz::mcherry(smu-1 introns)::gpr-1]) III; cxTi10816(he259[Peft-3::ph::co-egfp::co-lov::tbb-2(3'UTR)]) IV |  |
| **Figure 5-figure supplement 2** | AZ244 | unc-119(ed3) III; ruls57[Ppie-1::gfp::tbb-2 + unc119(+)] V | CGC |
|  | SV1815 | gpr-1(he266[∆gpr-1 -443 to 2039]) III; ruls57[Ppie-1::gfp::tbb-2 + unc119(+)] V |  |
|  | SV1816 | gpr-2 (he267[∆gpr-2 368 to 1953]) III; ruls57[Ppie-1::gfp::tbb-2 + unc119(+)] V |  |
|  | SV2075 | gpr-1(he301[glo-epdz::mcherry(smu-1 introns)::gpr-1]) III; ruls57[Ppie-1::gfp::tbb-2 + unc119(+)] V |  |
|  | SV2038 | gpr-1(he301[glo-epdz::mcherry(smu-1 introns)::gpr-1]), gpr-2(he311[Δ7 - 1212]) III; ruls57[Ppie-1::gfp::tbb-2 + unc119(+)] V |  |
| **Figure 5-figure supplement 3 a** | SV2085 | cxTi10816(he259[Peft-3::ph::co-egfp::co-lov::tbb-2(3'UTR)]) IV; ruls57[Ppie-1::gfp::tbb-2 + unc119(+)] V |  |
|  | SV2059 | gpr-1(he301[glo-epdz::mcherry(smu-1 introns)::gpr-1]), gpr-2(he311[Δ7 - 1212]) III; cxTi10816(he259[Peft-3::ph::co-egfp::co-lov::tbb-2(3'UTR)]) IV; ruls57[Ppie-1::gfp::tbb-2 + unc119(+)] V |  |
| **Figure 5-figure supplement 3 b** | SV2085 | cxTi10816(he259[Peft-3::ph::co-egfp::co-lov::tbb-2(3'UTR)]) IV; ruls57[Ppie-1::gfp::tbb-2 + unc119(+)] V |  |
|  | SV2059 | gpr-1(he301[glo-epdz::mcherry(smu-1 introns)::gpr-1]), gpr-2(he311[Δ7 - 1212]) III; cxTi10816(he259[Peft-3::ph::co-egfp::co-lov::tbb-2(3'UTR)]) IV; ruls57[Ppie-1::gfp::tbb-2 + unc119(+)] V |  |
|  |  |  |  |
| **Figure 6-figure supplement 1 a** | SV2085 | CxTi 10816(he259[Peft-3::PH::eGFP::LOV::tbb-2]) IV ; RuIs57 [ Ppie-1::Tub::GFP) V |  |
|  | SV2090 | dhc-1(he255[epdz::mcherry::dhc-1])/+ I; cxTi10816(he259[Peft-3::ph::co-egfp::co-lov::tbb-2(3'UTR)]) IV; ruls57[Ppie-1::gfp::tbb-2 + unc119(+)] V; |  |
| **Figure 6-figure supplement 1 b** | SV2085 | CxTi 10816(he259[Peft-3::PH::eGFP::LOV::tbb-2]) IV ; RuIs57 [ Ppie-1::Tub::GFP) V |  |
|  | SV2090 | dhc-1(he255[epdz::mcherry::dhc-1])/+ I; cxTi10816(he259[Peft-3::ph::co-egfp::co-lov::tbb-2(3'UTR)]) IV; ruls57[Ppie-1::gfp::tbb-2 + unc119(+)] V; |  |
| **Figure 6-figure supplement 1 c** | SV2095 | dhc-1(he255[epdz::mcherry::dhc-1]) I; ruls57[Ppie-1::gfp::tbb-2 + unc119(+)] V |  |
|  | SV2090 | dhc-1(he255[epdz::mcherry::dhc-1])/+ I; cxTi10816(he259[Peft-3::ph::co-egfp::co-lov::tbb-2(3'UTR)]) IV; ruls57[Ppie-1::gfp::tbb-2 + unc119(+)] V; |  |
|  |  |  |  |
| **Figure 6-figure supplement 2** | SV1673 | dhc-1(he255[epdz::mcherry::dhc-1]) I |  |
|  | SV2090 | dhc-1(he255[epdz::mcherry::dhc-1])/+ I; cxTi10816(he259[Peft-3::ph::co-egfp::co-lov::tbb-2(3'UTR)]) IV; ruls57[Ppie-1::gfp::tbb-2 + unc119(+)] V; |  |
|  | SV2171 | dhc-1(he255[epdz::mcherry::dhc-1])/+ I; dnc-1(cp271[dnc::co-mng::3xFlag]) IV; ruls57[Ppie-1::gfp::tbb-2 + unc119(+)] V |  |
|  | SV2172 | dhc-1(he255[epdz::mcherry::dhc-1])/+ I; cxTi10816(he259[Peft-3::ph::co-egfp::co-lov::tbb-2(3'UTR)]) IV; dnc-1(cp271[dnc::co-mng::3xFlag]) IV;  ruls57[Ppie-1::gfp::tbb-2 + unc119(+)] V; |  |
|  | SV2120 | lin-5(he330[lin-5::glo-epdz::mcherry(smu-1 introns)]) II; ruls57[Ppie-1::gfp::tbb-2 + unc119(+)] V; |  |
|  | SV2121 | lin-5(he330[lin-5::glo-epdz::mcherry(smu-1 introns)]) II; cxTi10816(he259[Peft-3::ph::co-egfp::co-lov::tbb-2(3'UTR)]) IV; ruls57[Ppie-1::gfp::tbb-2 + unc119(+)] V |  |
|  |  |  |  |
| **Figure 7-figure supplement 1** | SV2085 | cxTi10816(he259[Peft-3::ph::co-egfp::co-lov::tbb-2(3'UTR)]) IV; ruls57[Ppie-1::gfp::tbb-2 + unc119(+)] V |  |
|  | SV2120 | lin-5(he330[lin-5::glo-epdz::mcherry(smu-1 introns)]) II; ruls57[Ppie-1::gfp::tbb-2 + unc119(+)] V; |  |
|  | SV2121 | lin-5(he330[lin-5::glo-epdz::mcherry(smu-1 introns)]) II; cxTi10816(he259[Peft-3::ph::co-egfp::co-lov::tbb-2(3'UTR)]) IV; ruls57[Ppie-1::gfp::tbb-2 + unc119(+)] V |  |
| **Figure 7-figure supplement 2** | SV2085 | cxTi10816(he259[Peft-3::ph::co-egfp::co-lov::tbb-2(3'UTR)]) IV; ruls57[Ppie-1::gfp::tbb-2 + unc119(+)] V |  |
|  | SV2120 | lin-5(he330[lin-5::glo-epdz::mcherry(smu-1 introns)]) II; ruls57[Ppie-1::gfp::tbb-2 + unc119(+)] V; |  |
|  | SV2121 | lin-5(he330[lin-5::glo-epdz::mcherry(smu-1 introns)]) II; cxTi10816(he259[Peft-3::ph::co-egfp::co-lov::tbb-2(3'UTR)]) IV; ruls57[Ppie-1::gfp::tbb-2 + unc119(+)] V |  |
