# Supplementary material for: Optogenetic dissection of mitotic spindle positioning in vivo
Source: eLife. 2018 Aug 15;7:e38198. doi: 10.7554/eLife.38198 (PMC6214656; doi:10.7554/eLife.38198)
Supplement: Supplementary file 2. [file elife-38198-supp2.docx]

| **Supplementary Table 2: DNA oligo’s used in this study** | | | | | |
| --- | --- | --- | --- | --- | --- |
| **Used for allele** | **Code** | **Name** | **Purpose** | **PCR template** | **Sequence 5’ to 3’** |
|  |  |  |  |  |  |
| CxTi10816(he258[Peft-3::ph::co-egfp::co-lov::tbb-2(3'UTR)]) IV | oRS074 | GA Peft-3 PH R | clone pBSK HDR repair template | pVP019 | TCCTGGAGCCCGTGCATTTTTGAGCAAAGTGTTTCCCAACTGA |
|  | oRS075 | GA PH Peft-3 F |  | pVP019 | AAACACTTTGCTCAAAAATGCACGGGCTCCAGGATGAC |
|  | oRS076 | GA PH eGFP R |  | pLF035 | TTGGACATTCCCCCACCGCCCTTCTGCCGCTGGTCCATG |
|  | oRS077 | GA eGFP PH F |  | pLF035 | GGCAGAAGGGCGGTGGGGGAATGTCCAAGGGAGAGGAGCT |
|  | oLF082 | GA CxTi10816 fix F |  | pLF035 | AACAGTTTTGTCTTCCGACAGATGTGAGACGGAAAAATGGCCAAAATTCGAGATTTTGACTAAAATCAGTG |
|  | oLF138 | CxTi10816 eft-3 R |  | pLF035 | CAATAAAAGACCAAAGGTGCGTTTGGACGCAAGTACACGATTG |
|  | oLF139 | Eft-3 CxTi10816 F |  | pLF035 | TCGTGTACTTGCGTCCAAACGCACCTTTGGTCTTTTATTGTCAACT |
|  | oLF146 | Tbb-2 CxTi10816 R |  | pLF035 | AAATAGCAGGAAAATAAGAAATTTGGCCGTGTGTCACTAGTTGAGACTTTTTTCTTGGCGGC |
|  | oRS131 | CxTi10816 gRNA_F pJJR50 | ligate in pJJR50 sgRNA vector |  | /5PHOS/TCTTAGCTCAATCGTGTACTTGCG |
|  | oLF087 | CxTi10816 gRNA_R |  |  | /5PHOS/AAACCGCAAGTACACGATTGAGCT |
|  |  |  |  |  |  |
| rgs-7(he290[rgs-7::co-mcherry::co-epdz]) X | oLF233 | SEC mod mCh-ePDZ #1 F Bsu361 | clone pBSK HDR repair template | pLF056 | CCTCAGGagcatcgATGTCCAAGGGAGAGGAGGA |
|  | oLF234 | SEC mod mCh-ePDZ #2 R | modification of pDD082 (Addgene) | pLF056 | ctttccggattgaaagtacagattctcTCAGGTACGGTAGTTGATGGAGA |
|  | oLF284 | GA rgs-7 UTR RH SEC F |  | genomic DNA | CGACGACAAGCGTGATTACAAGGATGACGATGACAAGAGATGAgcaatcatattctcattttatgatctttttgt |
|  | oLF285 | GA rgs-7 UTR RH SEC R |  | genomic DNA | atttcacacaggaaacagctatgaccatgttatcgatttcaacaaaaatgtcaccatgtcaatcgaa |
|  | oLF282 | GA rgs-7 LH SEC F |  | genomic DNA | cccagtcacgacgttgtaaaacgacggccagtcgccggcaTTGAAAAAGGAGTTTTCCGATGAGAAC |
|  | oLF289 | GA rgs-7 LH SEC Rev wo linker |  | genomic DNA | CTCCCTTGGACATCGATGCTCCTGAGGCTCCCGATGCTCCTGACGGTGATGATGACGTGG |
|  |  |  |  |  |  |
| ttTi5605(he314[Ppie-1::glo-epdz::mcherry(smu-1)::tbb-2(3'UTR)]) II | oLF504 | GA Cx right ttTi5605 pBSK F | clone pBSK HDR repair template | genomic DNA | ccATCgaattatcaactatgtataatAAAGTTGCGGTACCgaatcactacgtgcgggatcat |
| epdz::mcherry | oLF505 | GA Cx right ttTi5605 xhoI cx left R |  | genomic DNA | cgaaatgtcctcctCTCGAGgattccatgatggtagcaaactcac |
|  | oLF506 | GA Cx left ttTi5605 XhoI cx rigth F |  | genomic DNA | accatcatggaatcCTCGAGaggaggacatttcgacaatgtcatataat |
|  | oLF507 | Ga Cx left ttTi5605 pBSK R |  | genomic DNA | AAGGGAACAAAAGCTGGAGCTCCACCGCGGTGGCGGCCGCggagttggtccaatttcgtgg |
|  | oLF508 | GA pie-1 enhancer ttTI5605 F |  | pAZ123 (Addgene) | cttgcacttaacgaagtgagtttgctaccatcatggaatcggaagtaaaatattgtaatattttccctaaatttcacttaaaaataa |
|  | oLF509 | long pie-1p 700 R |  | pAZ123 (Addgene) | CGGGAACATAAAATTCTGAGAATGCG |
|  | oLF510 | long pie-1p 652 F |  | pAZ123 (Addgene) | GCGCCAAAAACGTGGTGAAATA |
|  | oLF511 | GA long pie-p xhoI ttTi5605 R |  | pAZ123 (Addgene) | atttttcaaacattatatgacattgtcgaaatgtcctcctCTCGAGCTGGAAAAGAAAATTTGATTTTTAATTGTTTGGG |
|  | oRS316 | GA tbb-2 UTR mcherry (smu-1) R |  | pRS096 | aggattttgcatttatcTTACTTGTAAAGCTCATCCATTCCTCCG |
|  | oRS317 | GA mcherry (smu-1) tbb-2 UTR F |  | pRS053 | TGGATGAGCTTTACAAGTAAgataaatgcaaaatcctttcaagcattcc |
|  | oRS318 | GA ttTi5605 tbb-2 UTR R |  | pRS053 | tttttcaaacattatatgacattgtcgaaatgtcctcctCtgagacttttttcttggcggc |
|  | oRS336 | GA Ppie-1 long epdz GLO F v2 |  | pRS096 | TCCCAAACAATTAAAAATCAAATTTTCTTTTCCAGCaaaaATGCCAGAGCTCGGATTCTC |
|  |  |  |  |  |  |
| gpr-1(he301[glo-epdz::mcherry(smu-1 introns)::gpr-1]) III | oLF 465 | GA pGPR-1 glo-epdz R | clone pBSK HDR repair template | pLF063 | CTCCGACACCTCCGGAGATCGAGAATCCGAGCTCTGGCATtcgttttgcgaatgtggaaacttc |
|  | oLF 466 | GA pGPR-1 pbsk F |  | pLF063 | AAAACGACGGCCAGTGAATTGTAATACGACTCACTATAGGGCGAATTGGccagtttcatctcaagattctccatct |
|  | oLF 467 | GA GPR-1 mch(smu-1) F |  | pLF063 | CACCGGAGGAATGGATGAGCTTTACAAGggaggcggtgggATGGATGTCTCCTACTATGACGGT |
|  | oLF 468 | GA GPR-1 pbsk R |  | pLF063 | TAACCCTCACTAAAGGGAACAAAAGCTGGAGCTCCACCGCCGTAATAGGCGATGTGAACTGC |
|  | oLF482 | GLO ePDZ 1 F |  | pRS093 | ATGCCAGAGCTCGGATTCTC |
|  | oLF483 | GA MCH SMU-1 gpr-1 R |  | pRS093 | ACATCCATcccaccgcctccCTTGTAAAGCTCATCCATTCCTCCG |
|  | oVP283 | gpr-1 17 U6_gRNA_F | ligate in sgRNA vector |  | /5phos/aattATGGACGTCTCTTATTACGA |
|  | oVP284 | gpr-1 17 U6_gRNA_R |  |  | /5phos/aaacTCGTAATAAGAGACGTCCAT |
|  |  |  |  |  |  |
| gpr-2(he311[Δ7 - 1212]) III | oLF158 | gpr-2 5'UTR 99 gRNA F | ligate in sgRNA vector |  | /5phos/tcttgcttttgaaatgacgttggg |
|  | oLF159 | gpr-2 5'UTR 99 gRNA R |  |  | /5phos/aaaccccaacgtcatttcaaaagc |
|  | oLF160 | gpr-2 3'UTR 210 gRNA F |  |  | /5phos/tcttaaacagacaaaaatttcacg |
|  | oLF161 | gpr-2 3'UTR 210 gRNA R |  |  | /5phos/aaaccgtgaaatttttgtctgttt |
|  |  |  |  |  |  |
| gpr-1(he266[Δ -443 - + 2039]) III | oLF154 | gpr-1 5'UTR 62 gRNA F | ligate in sgRNA vector |  | /5phos/tcttagcaaaaataaaagaaatag |
|  | oLF155 | gpr-1 5'UTR 62 gRNA R |  |  | /5phos/aaacctatttcttttatttttgct |
|  | oLF156 | gpr-1 3'UTR 293 gRNA F |  |  | /5phos/tcttgagccggattttgaaattcg |
|  | oLF157 | gpr-1 3'UTR 293 gRNA R |  |  | /5phos/aaaccgaatttcaaaatccggctc |
|  |  |  |  |  |  |
| gpr-2(he267[Δ 368 - 1953]) III | oLF158 | gpr-2 5'UTR 99 gRNA F | ligate in sgRNA vector |  | /5phos/tcttgcttttgaaatgacgttggg |
|  | oLF159 | gpr-2 5'UTR 99 gRNA R |  |  | /5phos/aaaccccaacgtcatttcaaaagc |
|  | oLF160 | gpr-2 3'UTR 210 gRNA F |  |  | /5phos/tcttaaacagacaaaaatttcacg |
|  | oLF161 | gpr-2 3'UTR 210 gRNA R |  |  | /5phos/aaaccgtgaaatttttgtctgttt |
|  |  |  |  |  |  |
| lin-5(he330[lin-5::glo-epdz::mcherry(smu-1 introns)]) II | oRS382 | pBSK lin-5 CDS F | clone pBSK HDR repair template |  | GTGAATTGTAATACGACTCACTATAGGGCGAATTGGGCCGATAATGGACCAACAATTACC |
|  | oRS383 | ePDZ GLO lin-5 CDS R |  |  | GACACCTCCGGAGATCGAGAATCCGAGCTCTGGCATCTGCTTTTTGCTCGAAAAAGCAGA |
|  | oRS384 | lin-5 CDS ePDZ GLO F |  |  | TCGGGATTCGATTTTTTCTGCTTTTTCGAGCAAAAAGCAGATGCCAGAGCTCGGATTCTC |
|  | oRS385 | lin-5 UTR mCh smu-1 R |  |  | aaaaagttatattacaatgtatgaaagtatattTTACTTGTAAAGCTCATCCATTCCTCC |
|  | oRS386 | mCh smu-1 lin-5 UTR F |  |  | GGATGAGCTTTACAAGTAAaatatactttcatacattgtaatataactttttcacaacac |
|  | M13R | M13R |  |  | CAGGAAACAGCTATGACCATG |
|  | oRS428 | lin-5 2681 R |  |  | GATGGCGAATTCGTACTTCCAAC |
|  | oRS429 | lin-5 2755 F |  |  | GATTCGATTTTTTCTGCTTTTTCGAGC |
|  | oRS430 | lin-5 PAM disruption cloning fragment F |  |  | GGAAGTACGAATTCGCCATCtGCAGACGAGGAGAATATCAAGAAGTCCAAGAAAAAGAAtCGTCGaGATTCGATTTTTTCTGCTTTTTCG |
|  | oRS431 | lin-5 PAM disruption cloning fragment R |  |  | CGAAAAAGCAGAAAAAATCGAATCtCGACGaTTCTTTTTCTTGGACTTCTTGATATTCTCCTCGTCTGCaGATGGCGAATTCGTACTTCC |
|  |  |  |  |  |  |
| ric-8(he339[glo-epdz::mcherry(smu-1 introns)::ric-8) IV | oRS448 | ric-8 gRNA 0 F | ligate in sgRNA vector |  | tcttGGAATGTAGTTCTTCAGACA |
|  | oRS449 | ric-8 gRNA 0 R |  |  | aaacTGTCTGAAGAACTACATTCC |
|  | oRS450 | ric-8 gRNA 20 F |  |  | tcttccATGTCTGAAGAACTACAT |
|  | oRS451 | ric-8 gRNA 20 R |  |  | aaacATGTAGTTCTTCAGACATgg |
|  | oRS452 | ric-8 gRNA 21 F |  |  | tcttGAAAATCGACGCAATCAGGT |
|  | oRS453 | ric-8 gRNA 21 R |  |  | aaacACCTGATTGCGTCGATTTTC |
|  | oLF569 | GA PRIC-8 pbsk F | clone pBSK HDR repair template | Genomic DNA | GCCAGTGAATTGTAATACGACTCACTATAGGGCGAATTGGccgcacgtgcatgttgttta |
|  | oRS454 | oRS454 GA epdz glo ric-8p R |  | Genomic DNA | ggaattgcctgaaaatcatgaaaaattaattatttac |
|  | oRS455 | GA ric-8p epdz glo F |  | pRS121 | ctcgtaaataattaatttttcatgattttcaggcaattccATGCCAGAGCTCGGATTCTC |
|  | oRS456 | GA ric-8 cds mch smu-1 R |  | pRS121 | TCaGAATGTAGTTCTTCAGACATcccaccgcctccCTTGTAAAGCTCATCCATTCCTCCG |
|  | oRS457 | GA mch smu-1 ric-8 cds F |  | Genomic DNA | ACAAGggaggcggtgggATGTCTGAAGAACTACATTCtGACCTGATTGCGTCGATTTTCG |
|  | oLF571 | GA RIC-8 pbsk R |  | Genomic DNA | TAACCCTCACTAAAGGGAACAAAAGCTGGAGCTCCACCGCATTGACGACGTTTTTCGGCG |
|  | oRS458 | ric-8 check -993 F |  |  | acgttcttccgaaagttcgagc |
|  | oRS459 | GA ric-8 F |  |  | GTCTGAAGAACTACATTCtGACCTGATTG |
|  |  |  |  |  |  |
| dhc-1(he255[co-epdz::co-mcherry::dhc-1]) | oLF116 | Pdhc-1 -1119 Acc65I F | clone pBSK HDR repair template | pRS006 | aaaaGGTACCacgtggtgcttcgtgtataatttg |
|  | oLF137 | Pdhc-1 -28 HpaI R corr |  | pLF38 | aaaaGTTAACgtttagaatttgcaaaaagaacgaataaacgg |
|  | oVP315 | dhc-1 37 U6_gRNA_F | ligate in sgRNA vector |  | /5phos/aattgctgcggtttttaagtttgg |
|  | oVP316 | dhc-1 37 U6_gRNA_R |  |  | /5phos/aaacccaaacttaaaaaccgcagc |
|  |  |  |  |  |  |
| par-6(he322[par-6::gfp(smu-1 introns)::glo-lov] I |  | PAR-6_sgRNA_F | ligate in sgRNA vector |  | tcttGACGCAAATGATTCGGACAG |
|  |  | PAR-6_sgRNA_R |  |  | aaacCTGTCCGAATCATTTGCGTC |
|  |  | PAR-6_F2 | clone SEC HDR repair template | genomic DNA | acgttgtaaaacgacggccagtcgccggcactaggcgagcggaagttgaa |
|  |  | PAR-6_R2 |  |  | gatgctcctgaggctcccgatgctccGTCCTCTCCACTATCACTGTCATTTGCGTCGTGCT |
|  |  | PAR-6_F3 |  |  | CGTGATTACAAGGATGACGATGACAAGAGATGAaaaactcttttcagccatttttcc |
|  |  | PAR-6_R3 |  |  | ggaaacagctatgaccatgttatcgatttccccgaaattatgtcatttctggga |
|  | oLF484 | GFP smu-1 Bsu36I F |  | pRS101 | AAAAACCTCAGGAGCATCGTCCAAGGGAGAGGAGCTCT |
|  | oLF485 | glo-LOV Acc65I R |  |  | AAAAAGGTACCTGCGGCCGCTTAGACCCAAGTGTCGACGG |
